# Association between activities of daily living and depressive symptoms among older adults in China: evidence from the CHARLS

**DOI:** 10.3389/fpubh.2023.1249208

**Published:** 2023-11-16

**Authors:** Haixia Liu, Yang Ma, Lin Lin, Zekun Sun, Zeyu Li, Xinxin Jiang

**Affiliations:** ^1^School of Public Health and Management, Binzhou Medical University, Yantai, China; ^2^Periodicals Department, Binzhou Medical University, Yantai, China

**Keywords:** functional limitation, activities of daily living, depressive symptoms, Chinese older adults, BADL/IADL

## Abstract

**Objective:**

The limitation of activities of daily living (ADL) affects the mental health of older adults. We distinguished activities of daily living into basic activities of daily living (BADL) and instrumental activities of daily living (IADL) and aimed to explore the relationship between the two limitations and depressive symptoms among Chinese older adults by using nationally representative cross-sectional data.

**Methods:**

Data from the China Health and Retirement Longitudinal Study (CHARLS, wave 4) were used, and 9,789 older adults aged 60 years and above were screened. The 10-item Center for Epidemiologic Studies Depression (CES-D-10) scale was used to measure the depressive symptoms of older adults, and a 12-item scale for ADL was used to estimate functional limitations. Generalized linear mixed-effect models were employed to examine the relationship between BADL/IADL and depressive symptoms among older adults.

**Results:**

The prevalence of high-risk depression among older adults was 43.5%, and the rates of limitation in BADL and IADL were 19.02 and 25.29%, respectively. The prevalence of high-risk depression significantly differed among subgroups of smoking, drinking, chronic diseases, duration of sleep, having social activities or not, and the type of medical insurance. Older adults with limited BADL or IADL were at a higher risk of depression than those without limitations of BADL or IADL; BADL (OR_-adjusted_ = 2.71; 95% CI: 2.40–3.06) and IADL (OR_-adjusted_ = 2.68; 95% CI: 2.41–2.98) had various influences on the risk of depression in older adults.

**Conclusion:**

ADL was a related factor in the risk of depression among older adults in China. BADL and IADL had different effects on the risk of depression, suggesting that older adults with physical function limitations might be more likely to suffer from depression.

## 1. Introduction

The aging population is a considerable challenge worldwide (1). According to the World Health Organization, there were approximately 1 billion people aged 60 years and above in 2019, and this value will increase to 2.1 billion by 2050 (2). Aging is associated with impaired physical and cognitive health; it is also related to an increased risk of psychiatric problems such as depression (3, 4). A meta-analysis has shown that the prevalence of depression among older adults is 1.6% in the United States and 2.7% in China (5, 6). Depression among older adults has become an urgent and growing concern in China (7). Late-life depression increases the prevalence of physical illness, reduces the quality of life, causes many medical conditions, and exacerbates disability (8–10). From a clinical and public health perspective, the influencing factors of depressive symptoms among older adults should be explored to facilitate the early detection of depression and improve the quality of life (7). Some studies have indicated that gender, age, smoking, drinking, sleep, marital status, education, income, chronic diseases, and satisfaction of kinship needs may influence depressive symptoms among older adults (11–13). Some cross-sectional studies from Western Europe, South Africa, and Pakistan have reported that older adults with functional limitations or disabilities have a higher prevalence of depression than those without functional limitations or disabilities (14–16).

Activities of daily living (ADL) are widely recognized indicators of functional limitations in older adults and are often categorized into basic activities of daily living (BADL) and instrumental activities of daily living (IADL) (17). BADL and IADL reflect the activity ability of the older adults in two aspects. BADL refers to the most basic self-care ability that people must perform repeatedly every day to live independently; and IADL reflects an individual’s more complex ability to live independently and participate in social activities (18). Because different dimensions of functional limitation may be heterogeneous in association with depressive symptoms, the independent association between BADL/IADL and depressive symptoms among older adults should be analyzed. A study on adults aged 50 years and above from six low- and middle-income nations demonstrated a positive relationship between BADL/IADL disability and depression (19). Peng (20) included 3,951 adults over 45 years and showed that depressive symptoms aggravated the development of ADL disability. A longitudinal study on aging from Ireland found that depression is a risk factor for BADL/IADL disability among older adults (10). Previous study findings have suggested the relationship between ADL and depressive symptoms. However, most studies have focused on the effects of extreme ADL disability, and few studies have explored the early status of ADL limitations. In Malaysia, a cross-sectional study of 3,772 older adults (age ≥ 60 years) found that older adults with limitations in BADL are 2.58 times more likely to have depressive symptoms [adjusted odds ratio (aOR): 2.58, 95% CI: 2.01–3.32], while those with limitations in IADL are 1.68 times more likely to have depressive symptoms (aOR: 1.68, 95% CI: 1.32–2.14) (21). In the United States, a cross-sectional study of 207 Japanese-Americans (age ≥ 68 years) showed that older adults with high levels of functional status in IADL have lower odds of depressive symptoms (OR: 0.2, 95% CI: 0.09–0.50) (22). However, the functional status in BADL is not associated with depressive symptoms. Depending on socioeconomic and cultural factors, the relationship between depressive symptoms and functional limitations may vary in different countries. A study on older adults aged 60 years and above (*n* = 14,335) in three provinces in China showed that the effect of IADL limitation on depressive symptoms in the older adults is greater than that of BADL limitation (OR: 2.23 vs. 1.74) (23). Although the results are inconsistent, the effects of BADL and IADL on depressive symptoms may vary in older adults.

The results of some studies conducted in different regions and populations may be different, and broader evidence is needed to enrich research on this topic, especially some studies on Chinese older people via a national database. Therefore, this study aimed to analyze the influence of BADL/IADL on depressive symptoms and the risk of depression among older adults by using a nationally representative database of the China Health and Retirement Longitudinal Study (CHARLS). This study used a generalized linear mixed effects model (GLMM) and controlled relevant confounding factors to examine the following hypotheses:

*Hypothesis 1*: ADL is a significant influencing factor of depressive symptoms among older adults in China. The proportion of high-risk depression in older adults with ADL limitation is higher than that in older adults without ADL limitation.

*Hypothesis 2*: BADL and IADL have different impacts on depressive symptoms, especially BADL has greater effects on depressive symptoms than IADL among older adults.

## 2. Methods

### 2.1. Data and sample

Data were obtained from the fourth wave investigation of the China Health and Retirement Longitudinal Study (CHARLS) in 2018 via the CHARLS website.^1^ CHARLS is a survey of a nationally representative cohort of people who live in 23 Chinese provinces and are 45 years and older; this survey follows a randomized process using a multi-stage sampling method stratified by proportional probability to size sampling (24–26). The fourth wave of CHARLS was performed, and it included 19,816 adults aged 45 years and above from 150 counties/districts and 450 villages/urban communities (26). According to the definition of older adults in China, respondents aged 60 years and above were included as the research subjects. In this study, 11,055 individuals aged 60 years and above were included (the actual age of older adults was calculated according to the question “What is your actual date of birth?”). If the respondents did not complete or if they refused to answer the 10-item Center for Epidemiologic Studies Depression (CES-D-10) scale, they were removed from the study. Finally, 1,266 older adults were excluded, and 9,789 older adults were included in this study.

### 2.2. Measurements

#### 2.2.1. Measurement of depressive symptoms

In CHARLS, the CES-D-10 scale was used to estimate the risk of depressive symptoms. The CES-D-10 scale consists of eight negatively oriented items and two positively oriented items about the experiences of the participants in the past week. The negatively oriented items were “feeling bothered,” “having trouble concentrating,” “feeling depressed,” “feeling as though everything required effort,” “feeling fearful,” “having restless sleep,” “feeling lonely,” and “having difficulty getting going”; and the positively oriented items were “feeling hopeful” and “feeling happy.” Every item has the same selective answers: “rarely or none,” “some,” “occasionally,” and “most or all of the time.” The passively oriented items were scored from 0 for “rarely or none” to 3 for “most or all of the time,” while the positively oriented items were recorded scores in reverse. The scores ranged from 0 to 30; the higher the scores, the more severe the depressive symptoms and the higher the risk of depression. Previous studies on the CES-D-10 scale suggested that the cutoff point for depressive symptoms in older adults is 10 (20, 27–29). Therefore, a cutoff score of 10 was set to distinguish the participants with or without depressive symptoms (score ≥ 10: high-risk depression; score < 10: low-risk depression). The CES-D-10 has shown good effectiveness and reliability in Chinese (30, 31). In this study, Cronbach’s *α* of the CES-D-10 scale was 0.799, with good reliability, and the value of KMO was 0.889, with good validity.

#### 2.2.2. Measurement of activities of daily living

The limitations of activities of daily living (ADL) were measured in terms of basic activities of daily living (BADL) and instrumental activities of daily living (IADL), which are based on the Katz Index of Independence in ADL and the Lawton IADL Scale (17, 32–34). The Katz Index of Independence in ADL and the Lawton IADL Scale have been used in studies as independent indicators for assessing BADL and IADL in Chinese older adults (17, 35, 36). In CHARLS, BADL includes eating, dressing, bathing, transferring from a chair to a bed using the toilet, and defecating. IADL includes doing housework, cooking, making phone calls, shopping, taking medicine, and managing finances. All items have four options: “do it without difficulty,” “do it but with difficulty,” “do it with difficulty and need help,” and “cannot do it.” BADL and IADL were divided into two categories: unlimited BADL/IADL (the first options were selected in every item) and limited BADL or IADL (the last three options were chosen in any of the items). This scale displayed a great internal consistency in BADL/IADL from the 2018 CHARLS sample, with Cronbach’s *α* of 0.703 and 0.772, respectively. Validity analysis showed that the KMO values were 0.889 and 0.796 in BADL and IADL, respectively with great validity.

### 2.3. Statistical analysis

Because missing values are unavoidable in large cohorts, mean imputation can be used as an imputation method under the mechanism of missing at random (37, 38). The missing value rates for BADL and IADL variables are 0.98 and 1.00%, respectively. On the basis of cases with complete data, BADL and IADL were selected, and mean imputation was employed to fill in the missing data of each item. Descriptive statistics was used to analyze the basic characteristics of the participants, including the distribution of demographic characteristics, limitation of ADL, and depressive symptoms among older adults. A chi-square test was performed to compare the prevalence of high-risk depression in older adults between different sub-groups, and GLMMs were used to examine the association between BADL/IADL and depressive symptoms. A GLMM is a multilevel model with fixed and random effects. The mathematical formula for GLMM is expressed as follows:


(1) $$\eta =g\left[E\left(y\right)\right]=X\beta +Z\mu +\varepsilon \text{,}$$

where *g*(*y*) is the link function, *β* is the fixed effects, and *μ* is the random effects. The definition and assignment of variables are presented in Table 1.

**Table 1:** Definition and assignment of variables in GLMM analysis.

Variable settings	Variables	Value of variables
Dependent variable	Depressive symptoms	High-risk depression = 1, low-risk depression = 0
Fixed effect variables	BADL/IADL	Limited = 1, unlimited = 0
	Sex	male = 1, female = 2
	Age (years)	60–64 = 1, 65–74 = 2, ≥75 = 3
	Household registration	Urban = 1, rural = 2
	Marital status	Married and cohabiting = 1, married but separated = 2, divorced = 3, widowed = 4, never married = 5
	Ethnic groups	Han = 1, non-Han = 2
	Education	illiteracy = 1, primary school and below = 2, elementary school and above = 3
	Income	low = 1, middle = 2, high = 3
	Type of medical insurance	Urban employees = 1, urban and rural residents = 2, free medical care = 3, commercial insurance and others = 4
	Smoking	Smoking = 1, quitting = 2, never smoking = 3
	Drinking	Drinking = 1, quitting = 2, never drinking = 3
	Chronic diseases	No chronic disease = 1, single chronic disease = 2, two or more chronic diseases = 3
	Duration of sleep (hour)	<6 = 1, 6–9 = 2, >9 = 3
	Having social activities or not	no = 0, yes = 1
	Life satisfaction	Dissatisfied = 0, satisfied = 1
	Health satisfaction	Dissatisfied = 0, satisfied = 1
	Marital satisfaction	Dissatisfied = 0, satisfied = 1
	Child relationship satisfaction	Dissatisfied = 0, satisfied = 1
	Self-reported health	Very good = 1, good = 2, fair = 3, poor = 4, very poor = 5
Random effect variables	First level	Individual
	Second level	Household
	Third level	Community

In this study, the dependent variable (*Y*) was a categorical variable that followed a binomial distribution; as such, “logit” was chosen as the link function. Unadjusted models (models 1, 2, and 3) and adjusted models (models 4, 5, and 6) were used to examine the association between BADL/IADL and depressive symptoms. The parameter was calculated using the Laplace approximation method. A GLMM was created using the “glmer” function of package “lme4” in R4.2.2 software, and results were expressed as unadjusted odds ratios (OR_-unadjusted_), adjusted odds ratios (OR_-adjusted_), and their 95% confidence intervals (95% CI).

## 3. Results

The characteristics of the participants are shown in Table 2. A total of 9,789 older adults were included in this study; among them, 4,779 (48.82%) were men and 5,010 (51.18%) were women. Of all participants, 30.74% were 60–64 years old, 43.65% were 65–74 years old, and 25.61% were 85 years old and above. Furthermore, 73.41% of older adults were from rural areas, 54.40% had two or more types of chronic diseases, and 46.50% did not participate in any social activities within 1 month before the investigation. As for the satisfaction of health, life, children’s relationships, or marriage, 65.25% of the older adults were satisfied with their health, 80.70% were satisfied with their life, 94.46% were satisfied with their children’s relationship, and 78.94% were satisfied with their marriage. Additionally, 43.5% of older adults had high-risk depression, and 19.02 and 25.29% of the participants were limited in BADL and IADL, respectively.

**Table 2:** Characteristics of the participants (*N*, %).

Variable	*N* (%)	Variable	*N* (%)
Sex		Type of insurance	
Male	4,779 (48.82)	Urban employees	1,320 (13.48)
Female	5,010 (51.18)	Urban and rural residents	7,504 (76.66)
Age (year)		Free medical care	617 (6.30)
60–64	3,009 (30.74)	Commercial insurance and others	348 (3.56)
65–74	4,273 (43.65)	Income	
≥75	2,507 (25.61)	Low	3,728 (38.08)
Household registration		Middle	4,858 (49.63)
Urban	2,603 (26.59)	High	1,203 (12.29)
Rural	7,186 (73.41)	Health satisfaction	
Marital status		Dissatisfied	3,402 (34.75)
Married and cohabiting	7,287 (74.44)	Satisfied	6,387 (65.25)
Married but separated	365 (3.73)	Life satisfaction	
Divorced	94 (0.96)	Dissatisfied	1889 (19.30)
Widowed	1974 (20.17)	Satisfied	7,900 (80.70)
Never married	69 (0.70)	Child relationship satisfaction	
Ethnic groups		Dissatisfied	453 (4.63)
Han	9,088 (92.84)	Satisfied	9,247 (94.46)
Non-Han	701 (7.16)	No children	89 (0.91)
Education		Marital satisfaction	
Illiteracy	3,103 (31.70)	Dissatisfied	846 (8.64)
Primary school and below	4,256 (43.48)	Satisfied	7,727 (78.94)
Elementary school and above	2,430 (24.82)	No spouse	1,216 (12.42)
Smoking status		Self-reported health	
Smoking	2,794 (28.54)	Very good	888 (9.07)
Quitting	1,313 (13.41)	Good	1,000 (10.22)
Never smoking	5,682 (58.04)	Fair	5,128 (52.39)
Drinking status		Bad	2,149 (21.95)
Drinking	2,502 (25.56)	Very bad	624 (6.37)
Quitting	741 (7.57)	BADL	
Never drinking	6,534 (66.75)	Not limited	7,927 (80.98)
Chronic diseases		Limited	1862 (19.02)
No chronic disease	2088 (21.33)	IADL	
Single chronic disease	2,376 (24.27)	Not limited	7,313 (74.71)
Two or more chronic diseases	5,325 (54.40)	Limited	2,476 (25.29)
During of sleep (hour)		Risk of depression	
<6	3,317 (33.88)	Low risk	5,531 (56.5)
6–9	6,055 (61.86)	High risk	4,258 (43.5)
>9	417 (4.26)		
Having social activities or not			
No	4,552 (46.50)		
Yes	5,237 (53.50)		

The prevalence of high-risk depression significantly differed among the groups in terms of smoking, drinking, chronic diseases, sleep duration, having social activities or not, and type of insurance (Table 3). The prevalence of high-risk depression was 68.8% in the older adults with limited BADL and 66.0% in the older adults with limited IADL. Significant differences were observed between BADL/IADL and depressive symptoms in the whole sample.

**Table 3:** Comparison of the prevalence of high-risk depression among older adults (*N*/%).

Variable	Depression		*χ*^2^	Variable	Depression		*χ*^2^
	Low risk	High risk			Low risk	High risk	
Sex				Having social activities or not			
Male	2,721 (56.9)	2058 (43.1)	0.75	No	2,416 (53.1)	2,136 (46.9)	40.41**
Female	2,809 (56.5)	2,201 (43.5)		Yes	3,114 (59.5)	2,123 (43.5)	
Age (year)				Type of insurance			
60–64	1714 (57.0)	1,295 (43.0)	0.46	Urban employees	849 (64.3)	471 (35.7)	40.83**
65–74	2,400 (56.2)	1873 (43.8)		Urban and rural residents	4,125 (55.0)	3,379 (45.0)	
≥75	1,416 (56.5)	1,091 (43.5)		Free medical care	360 (58.3)	257 (41.7)	
Household registration				Commercial insurance and others	196 (56.3)	152 (43.7)	
Urban	1,486 (57.1)	1,117 (42.9)	0.66	Income			
Rural	4,404 (56.3)	3,142 (43.7)		Low	1989 (53.4)	1739 (46.6)	28.51**
Marital status				Middle	2,806 (57.8)	2052 (42.2)	
Married and cohabiting	4,112 (56.4)	3,175 (43.6)	0.66	High	735 (61.1)	468 (38.9)	
Married but separated	213 (58.4)	152 (41.6)		Health satisfaction			
Divorced	54 (57.4)	40 (42.6)		Dissatisfied	1944 (57.1)	1,458 (42.9)	0.9
Widowed	1,111 (56.3)	863 (43.7)		Satisfied	3,586 (56.1)	2,801 (43.9)	
Never married	40 (58.0)	29 (42.0)		Life satisfaction			
Ethnic groups				Dissatisfied	1,061 (56.2)	828 (43.8)	0.1
Han	5,130 (56.4)	3,958 (43.6)	0.1	Satisfied	4,469 (56.6)	3,431 (43.4)	
Non-Han	400 (57.1)	301 (42.9)		Child relationship satisfaction			
Education				Dissatisfied	268 (59.2)	185 (40.8)	1.75
Illiteracy	1733 (55.8)	1,370 (44.2)	2.68	Satisfied	5,209 (56.3)	4,038 (43.7)	
Primary school and below	2,390 (56.2)	1866 (43.8)		No children	53 (59.6)	36 (43.5)	
Elementary school and above	1,407 (57.9)	1,023 (42.1)		Marital satisfaction			
Smoking status				Dissatisfied	486 (57.4)	360 (42.6)	0.43
Smoking	1714 (61.3)	1,080 (38.7)	60.52**	Satisfied	4,363 (56.5)	3,364 (43.5)	
Quitting	794 (60.5)	519 (39.5)		No spouse	681 (56.0)	535 (44.0)	
Never smoking	3,022 (53.2)	2,660 (46.8)		Self-reported health			
Drinking or not				Very good	487 (54.8)	401 (45.2)	7.68
Drinking	1,597 (63.8)	905 (36.2)	80.32**	Good	561 (56.1)	439 (43.9)	
Quitting	438 (59.1)	303 (40.9)		Fair	2,902 (56.6)	2,226 (43.4)	
Never drinking	3,489 (53.4)	3,045 (46.6)		Bad	1,197 (55.7)	952 (44.3)	
Chronic diseases				Very bad	383 (61.4)	241 (38.6)	
No chronic disease	1,496 (71.6)	592 (28.4)	399.59**	BADL			
Single chronic disease	1,492 (62.8)	884 (37.2)		Not limited	4,949 (62.4)	2,978 (37.6)	598.30**
Two or more chronic disease	2,542 (47.7)	2,783 (52.3)		Limited	581 (31.2)	1,281 (68.8)	
During of sleep (hour)				IADL			
<6	1,362 (41.1)	1955 (58.9)	487.42**	Not limited	4,689 (64.1)	2,624 (35.9)	684.23**
6 ~ 9	3,911 (64.6)	2,144 (35.4)		Limited	841 (34.0)	1,635 (66.0)	
>9	257 (61.6)	160 (38.4)					

(1) ADL, activities of daily life; BADL, basic activities of daily living; IADL, instrumental activities of daily living. (2) *p < 0.05 and **p < 0.01.

In the unadjusted model combining BADL and IADL, BADL (OR_-unadjusted_ = 2.31, 95% CI: 2.04–2.62) and IADL (OR_-unadjusted_ = 2.42, 95% CI: 2.17–2.71) were associated with the prevalence of high-risk depression in older adults (Table 4). BADL (OR_-unadjusted_ = 3.58, 95% CI: 3.19–4.03) and IADL (OR_-unadjusted_ = 3.39, 95% CI: 3.06–2.74) were separately associated with high-risk depression (model 2 and model 3). The results of the unadjusted models (models 1, 2, and 3) showed that older adults with limited BADL/IADL were more likely to experience depressive symptoms than older adults without limited BADL/IADL.

**Table 4:** Association of activities of daily living and depressive symptoms among older adults (China, 2018–2019).

Variables	Classification	Model 1		Model 2		Model 3	
		OR_-unadjusted_	95% CI	OR_-unadjusted_	95% CI	OR_-unadjusted_	95% CI
–	(Intercept)	0.51**	0.48,0.54	0.59**	0.55,0.63	0.55**	0.51,0.58
BADL	Unlimited	Ref	–	Ref	–	–	–
	Limited	2.31**	2.04,2.62	3.58**	3.19,4.03	–	–
IADL	Unlimited	Ref	–	–	–	Ref	–
	Limited	2.42**	2.17,2.71	–	–	3.39**	3.06,3.74

(1) OR, odds ratio; CI, confidence interval; Ref, reference. (2) *p < 0.05 and **p < 0.01.

After controlling for age, sex, household registration, marital status, education, ethnic groups, income, type of medical insurance, smoking, drinking, having social activities or not, duration of sleep, chronic diseases, self-reported health, health satisfaction, life satisfaction, marital satisfaction, and child relationship satisfaction, we found that the combined BADL (OR_-adjusted_ = 1.92; 95% CI: 1.68–2.18) and IADL (OR_-adjusted_ = 2.10, 95% CI: 1.87–2.36) were associated with the prevalence of high-risk depression in older adults. In model 5 and 6, BADL (OR_-adjusted_ = 2.71, 95% CI: 2.40–3.06) and IADL (OR_-adjusted_ = 2.68, 95% CI: 2.41–2.98) were independently associated with depressive symptoms (Table 5). The comparison of the results between the adjusted model and unadjusted model, showed that the prevalence of high-risk depression decreased from 3.58 times to 2.71 times among the older adults with limited BADL, higher than those without limited BADL after controlling for other confounding variables. This change was also observed in IADL, which decreased from 3.39 to 2.68 times.

**Table 5:** Association of daily living and depressive symptoms and its domains among older adults (China, 2018–2019).

Variables	Classification	Model 4		Model 5		Model 6	
		OR_-adjusted_	95% CI	OR_-adjusted_	95% CI	OR_-adjusted_	95% CI
–	(Intercept)	0.38**	0.23,0.64	0.43**	0.26,0.71	0.40**	0.24,0.65
BADL	Unlimited	Ref	–	Ref	–	–	–
	Limited	1.92**	1.68,2.18	2.71**	2.40,3.06	–	–
IADL	Unlimited	Ref	–	–	–	Ref	–
	Limited	2.10**	1.87,2.36	–	–	2.68**	2.41,2.98
Sex	Male	Ref	–	Ref		Ref	–
	Female	1.06	0.96,1.17	1.04	0.95,1.15	1.07	0.97,1.18
Age	60–64	Ref	–	Ref	–	Ref	–
	65–74	1.07	0.96,1.19	1.07	0.96,1.19	1.05	0.94,1.17
	≥75	1.01	0.89,1.15	1.02	0.90,1.17	0.99	0.87,1.13
Household registration	Urban	Ref	–	Ref	–	Ref	–
	Rural	1.02	0.90,1.16	1.01	0.89,1.14	1.02	0.90,1.15
Marital status	Married and cohabiting	Ref	–	Ref	–	Ref	–
	Separated	1.03	0.81,1.31	1.01	0.80,1.29	1.02	0.80,1.29
	Divorced	1.00	0.62,1.62	0.99	0.61,1.60	1.05	0.65,1.69
	Widowed	1.00	0.84,1.19	1.00	0.84,1.19	0.99	0.83,1.17
	Never married	1.03	0.51,2.08	0.96	0.48,1.93	0.99	0.49,1.99
Education	Illiteracy	Ref	–	Ref	–	Ref	–
	Primary school and below	1.03	0.91,1.15	1.01	0.90,1.13	1.03	0.92,1.15
	Elementary school and above	0.96	0.83,1.11	0.95	0.82,1.09	0.96	0.83,1.10
Ethnic groups	Han	Ref	–	Ref	–	Ref	–
	Non-Han	0.99	0.81,1.21	0.98	0.80,1.21	1.00	0.81,1.22
Income	Low	Ref	–	Ref	–	Ref	–
	Middle	0.93	0.84,1.02	0.92	0.84,1.02	0.93	0.85,1.03
	High	0.98	0.83,1.16	0.96	0.82,1.13	0.99	0.84,1.17
Type of insurance	Urban employees	Ref	–	Ref	–	Ref	–
	Urban and rural residents	1.20*	1.03,1.40	1.20*	1.02,1.40	1.22*	1.04,1.42
	Free medical care	1.21	0.97,1.51	1.19	0.95,1.48	1.20	0.96,1.50
	Commercial insurance and others	1.20	0.91,1.59	1.18	0.89,1.57	1.22	0.93,1.62
Smoking	Smoking	Ref	–	Ref	–	Ref	–
	Quitting	0.82**	0.71,0.95	0.82**	0.71,0.95	0.83*	0.72,0.97
	Never smoking	1.17**	1.05,1.31	1.19**	1.07,1.33	1.18**	1.05,1.32
Drinking	Drinking	Ref	–	Ref	–	Ref	–
	Quitting	1.20	1.00,1.45	1.20	0.99,1.45	1.21	1.00,1.45
	Never drinking	1.15*	1.03,1.29	1.21**	1.08,1.36	1.16**	1.04,1.31
Having social activities or not	No	Ref	–	Ref	–	Ref	–
	Yes	0.91*	0.83,1.00	0.88**	0.80,0.96	0.89*	0.82,0.98
	<6 h	Ref	–	Ref	–	Ref	–
During of sleep	6–9 h	0.46	0.42,0.50	0.45	0.40,0.49	0.44	0.40,0.49
	>9 h	0.42	0.33,0.53	0.42	0.34,0.53	0.41	0.33,0.52
Chronic diseases	No chronic disease	Ref	–	Ref	–	Ref	–
	Single chronic disease	1.34**	1.17,1.54	1.39**	1.21,1.59	1.37**	1.20,1.57
	Two or more chronic diseases	1.99**	1.76,2.25	2.15**	1.90,2.43	2.12**	1.88,2.40
Self-reported health	Very good	Ref	–	Ref	–	Ref	–
	Self-reported health	0.97	0.92,1.02	0.98	0.93,1.03	0.97	0.93,1.03
Health satisfaction	Dissatisfied	Ref	–	Ref	–	Ref	–
	Satisfied	1.03	0.91,1.16	1.02	0.91,1.16	1.04	0.92,1.17
Life satisfaction	Dissatisfied	Ref	–	Ref	–	Ref	–
	Satisfied	0.96	0.84,1.10	0.97	0.84,1.11	0.95	0.83,1.09
	Dissatisfied	Ref	–	Ref	–	Ref	–
Marital satisfaction	Satisfied	1.01	0.85,1.20	0.99	0.83,1.18	1.03	0.86,1.22
	No spouse	1.08	0.84,1.39	1.06	0.82,1.36	1.11	0.86,1.43
Child relationship satisfaction	Dissatisfied	Ref	–	Ref	–	Ref	–
	Satisfied	1.10	0.88,1.38	1.12	0.90,1.40	1.08	0.87,1.35
	No children	1.05	0.55,2.03	1.09	0.57,2.10	1.03	0.54,1.97

(1) All models included the individual, family, and community factors as random effects and adjusted for confounding factors of sex, age, household registration, marital status, education, ethnic groups, income, type of insurance, smoking, drinking, having social activities or not, during of sleep, chronic diseases, self-reported health, health satisfaction, life satisfaction, marital satisfaction, child relationship satisfaction. (2) OR, odds ratio; CI, confidence interval; Ref, reference. (3) *p < 0.05 and **p < 0.01.

## 4. Discussion

This study found that Chinese older adults with functional limitations in BADL/IADL, especially in BADL, were independently associated with depressive symptoms. Moreover, the variables of smoking, drinking, chronic diseases, sleep duration, social activities, and type of insurance may be associated with depressive symptoms among older adults in China.

The prevalence of high-risk depression among older adults in China was 43.5%, which was significantly more severe than that in the United States (24.0%), South Korea (16.1%), Singapore (7.8%), Sri Lanka (27.8%), and Taiwan (18.9%) (19). The prevalence of depressive symptoms varies widely not only because of differences in population, environment, culture, and economy of studies but also because of differences in measurement methods and criteria. Therefore, various situations should be considered when making cross-country comparisons. Because of the coexistence of BADL/IADL and depressive symptoms, the prevalence of limited BADL/IADL and its relationship with depressive symptoms among older adults should be investigated. Our findings revealed that limited BADL and IADL among older Chinese had a prevalence of 19.02, and 25.29%, respectively. In Canada, the prevalence of BADL or IADL limitation is 10% (39). By comparison, the prevalence in this study is also higher than BADL limitation (18%) and IADL limitation (17%) in the United States (40, 41). Therefore, the situation of functional limitations was not optimistic among older adults.

This study examined the association between BADL/IADL and depressive symptoms based on GLMM, which included the random effects of individual, family, and community factors. The result revealed that older adults with limited BADL/IADL were more likely to experience depressive symptoms than those without limited BADL/IADL, even after the confounding variables were controlled. This result confirmed our hypothesis and was supported by previous studies that the functional limitations of BADL/IADL may be a contributing factor to depressive symptoms (20, 28, 29, 42–45). Furthermore, older adults with limited BADL had a stronger association with depressive symptoms with limited IADL in this study. This result confirmed our hypothesis 2 and was supported by previous studies (21, 46). Similarly, in a cross-sectional study on 522 older adults from Dalian, BADL is more strongly associated with depressive symptoms (46). A cross-sectional study in Malaysia also supported our finding that older adults with limitations in BADL are more likely to have depressive symptoms than those in IADL (aOR: 2.58 vs. 1.68) (21). However, among older adults in the United States, only IADL and not BADL, can significantly predict depression (21). For these older Americans, maintaining valuable social roles is important in preventing depression because limitations in IADL may prevent older adults from fulfilling social roles and reduce their opportunities to participate in daily activities (such as shopping, taking public transportation, and walking around the community). In this study, on Chinese older adults, BADL was more sensitive than IADL in terms of identifying depressive symptoms. Inconsistencies in results may be due to differences in cultural background, economic development levels, health services, study populations, and analytic methods.

The relationship between BADL/IADL and depressive symptoms in older adults is complex, and they may have a mutual, potentially spiraling association (47). It is difficult to explain their specific mechanism clearly. Generally, older adults people initially experience limitations in IADL and later in BADL. The limitation of IADL is accompanied by a decrease in goal-oriented behavior and an increase in negative emotions. Limitations in BADL are related to the loss of physical function (such as loss of lower body strength or impaired mobility), which affects an individual’s mood (48). Furthermore, it can restrict one’s activities and decrease social activities, thus leading to depression (47, 49). The worsening of BADL is also associated with the loss of physical function, such as loss of lower body strength or impaired mobility, which can influence an individual’s mood (50). People with lower BADL levels may be more vulnerable to mental health deterioration than people with lower IADL levels because the former are unable to care for themselves. Additionally, a previous study hypothesized that limited ADL and depression may have similar hormonal and metabolic pathways; specifically, depression is related to high cortisol levels (51), and physical activity can reduce cortisol levels in people with depression (52). A previous study found that work may partly improve the physical condition of older people and increase their sense of self-worth (29). However, people aged 60 years and above in China basically retire and stop working, possibly leading to more feelings of frustration, irritability, depression, or sadness. Additionally, Chinese older adults are less likely to receive socialized care than in developed countries, and they mostly rely on home-based care to limit costs and precarious long-term care (46). Therefore, maintaining BADL’s ability to live an independent life is a priority for Chinese older adults.

In the present study, significant differences were observed in the prevalence of depressive symptoms among the variable groups of smoking status, drinking status, chronic diseases, sleep duration, having social activities or not, and type of insurance. We could consider that smoking, drinking, and lack of sleep may affect depressive symptoms. Therefore, minimizing these unhealthy lifestyle habits and behaviors may play a primary role in promoting health education for depression among older adults. Another important finding is that older adults with chronic diseases or without social activity are more likely to experience depressive symptoms. Li (32) revealed that somatic-mental multi-morbidity is associated with a higher risk of ADL/IADL disability. In Canada, people with multi-morbidity and mental illness have an increased risk of developing functional limitations compared with those who do not have mental illness (39). Older adults with chronic diseases are given more attention and timely interventions, which can effectively alleviate the impact of limited ADL on depressive symptoms. In addition, older people are encouraged to participate in social interaction activities. When these older adults feel socially supported, they feel less lonely and depressed, thereby reducing the risk of depression. Tian (53) also proposed that functional disability affected depressive symptoms, and social support could moderate this effect.

This study used data from a national survey, and the sample is representative. The results of this study provide evidence for understanding the association of BADL/IADL with depressive symptoms in Chinese older adults. However, there are still some limitations in this study. First, because it is a cross-sectional study, the causal relationship between BADL/IADL and depressive symptoms among older adults is difficult to obtain. Thus, prospective cohort studies should be conducted to validate the causality between BADL/IADL. Second, functional limitation and depressive symptoms are derived from self-reported screening rather than clinical diagnostic methods, potentially causing bias against valid values. Finally, the CES-D-10 scale scores were converted into categorized data, and the mean imputation of missing values was performed for BADL and IADL; however, this approach might underestimate the association between depressive symptoms and BADL/IADL. Therefore, two sensitivity analyses were performed to test the robustness of the results. (1) The CES-D-10 score of the dependent variable was used as quantitative data for GLMM regression analysis. (2) After the missing values of BADL/IADL were removed, regression analysis was also conducted, and the results of both sensitivity analyses remained reliable (Supplementary material).

## 5. Conclusion

This study indicated that limitation in BADL/IADL was independently and positively associated with depressive symptoms among older adults, especially IADL. Additionally, the factors of smoking, drinking, chronic diseases, sleep duration, social activities, and type of insurance were associated with depressive symptoms. In future, studies should focus on the prevention of depressive symptoms in people with low levels of BADL/IADL, which may be crucial in reducing the risk of depression.

## Data availability statement

Publicly available datasets were analyzed in this study. This data can be found at: https://charls.ccer.edu.cn/charls/.

## Ethics statement

The studies involving humans were approved by Institutional Review Board (IRB) of Peking University. The studies were conducted in accordance with the local legislation and institutional requirements. Written informed consent for participation was not required from the participants or the participants' legal guardians/next of kin because The IRB approval number for the main household survey including anthropometrics is IRB00001052-11015, and the IRB approval number for biomarker collection is IRB00001052-11014.Written informed consent for participation was not required for this study in accordance with the national legislation and the institutional requirements.

## Author contributions

HL, LL, and YM: conceptualization. LL and YM: methodology. YM and ZL: software. YM: formal analysis and writing of the original draft. YM, ZS, XJ, and HL: review and modification. All authors contributed to the article and approved the submitted version.

## Footnotes

^1^http://www.charls.pku.edu.cn/en
