# Supplementary material for: Association between activities of daily living and depressive symptoms among older adults in China: evidence from the CHARLS
Source: Front Public Health. 2023 Nov 16;11:1249208. doi: 10.3389/fpubh.2023.1249208 (PMC10687586; doi:10.3389/fpubh.2023.1249208)
Supplement: Supplementary file 1 [file Table_1.DOCX]

**Supplementary materials**

**Table S1** Association of daily living and scores of CES-D and its domains among older adults (N=9789)

| Variables | Classification | Model 1 | | Model 2 | | Model 3 | |
| --- | --- | --- | --- | --- | --- | --- | --- |
|  |  | *OR_-unadjusted_* | 95% *CI* | *OR_-unadjusted_* | 95% *CI* | *OR_-unadjusted_* | 95% *CI* |
| - | (Intercept) | 6.07** | 5.90,6.24 | 0.59** | 0.56,0.63 | 0.56** | 0.52,0.59 |
| BADL | Unlimited | *Ref* | - | *Ref* | - | - | - |
|  | Limited | 1.45** | 1.38,1.52 | 3.54** | 3.15,3.98 |  |  |
| IADL | Unlimited | *Ref* | - | - | - | *Ref* | - |
|  | Limited | 1.46** | 1.40,1.53 |  |  | 3.35** | 3.03,3.71 |

Note: *OR*: odds ratio; *CI*: confidence interval; *Ref*: reference. (2) **P*<0.05; ***P*<0.01.

**Table S2** Association of daily living and scores of CES-D and its domains among older adults (N=9789)

| Variables | Classification | Model 4 | | Model 5 | | Model 6 | |
| --- | --- | --- | --- | --- | --- | --- | --- |
|  |  | *OR_-adjusted_* | 95% *CI* | *OR_-adjusted_* | 95% *CI* | *OR_-adjusted_* | 95% *CI* |
| - | (Intercept) | 7.12** | 6.26,8.09 | 7.30** | 6.41,8.31 | 7.22** | 6.35,8.22 |
| BADL | Unlimited | *Ref* | - | *Ref* | - | - | - |
|  | Limited | 1.27** | 1.22,1.32 | 1.43** | 1.38,1.48 | - | - |
| IADL | Unlimited | *Ref* | - | - | - | *Ref* | - |
|  | Limited | 1.29** | 1.24,1.34 | - | - | 1.42** | 1.37,1.47 |
| Sex | Male | *Ref* | - | *Ref* | *-* | *Ref* | - |
|  | Female | 1.02 | 0.99,1.05 | 1.02 | 0.99,1.05 | 1.02 | 0.99,1.05 |
| Age | 60-64 | *Ref* | - | *Ref* | - | *Ref* | - |
|  | 65-74 | 1.02 | 0.98,1.05 | 1.02 | 0.98,1.05 | 1.01 | 0.98,1.04 |
|  | ≥75 | 1.01 | 0.97,1.05 | 1.02 | 0.98,1.06 | 1.00 | 0.96,1.04 |
| Household registration | Urban | *Ref* | - | *Ref* | - | *Ref* | - |
|  | Rural | 1.00 | 0.96,1.04 | 0.99 | 0.95,1.03 | 1.00 | 0.96,1.04 |
| Marital status | Married and cohabiting | *Ref* | - | *Ref* | - | *Ref* | - |
|  | Separated | 1.04 | 0.97,1.12 | 1.03 | 0.96,1.11 | 1.03 | 0.96,1.11 |
|  | Divorced | 0.97 | 0.84,1.12 | 0.96 | 0.83,1.12 | 0.98 | 0.85,1.14 |
|  | Widowed | 1.00 | 0.95,1.05 | 1.00 | 0.95,1.05 | 1.00 | 0.95,1.05 |
|  | Never married | 1.01 | 0.82,1.26 | 0.99 | 0.80,1.23 | 1.00 | 0.80,1.24 |
| Education | Illiteracy | *Ref* | - | *Ref* | - | *Ref* | - |
|  | Primary school and below | 1.02 | 0.99,1.06 | 1.02 | 0.98,1.05 | 1.02 | 0.99,1.06 |
|  | Elementary school and above | 1.01 | 0.97,1.05 | 1.01 | 0.96,1.05 | 1.01 | 0.96,1.05 |
| Ethnic groups | Han | *Ref* | - | *Ref* | - | *Ref* | - |
|  | Non-Han | 0.99 | 0.93,1.06 | 0.99 | 0.93,1.06 | 1.00 | 0.93,1.06 |
| Income | Low | *Ref* | - | *Ref* | - | *Ref* | - |
|  | Middle | 0.98 | 0.95,1.01 | 0.98 | 0.95,1.01 | 0.98 | 0.95,1.01 |
|  | High | 1.00 | 0.95,1.05 | 1.00 | 0.95,1.05 | 1.00 | 0.95,1.06 |
| Type of insurance | Urban employees | *Ref* | - | *Ref* | - | *Ref* | -- |
|  | Urban and rural residents | 1.06* | 1.01,1.11 | 1.06* | 1.01,1.11 | 1.07** | 1.02,1.12 |
|  | Free medical care | 1.08* | 1.01,1.16 | 1.07* | 1.00,1.15 | 1.08* | 1.01,1.16 |
|  | Commercial insurance and others | 1.05 | 0.97,1.15 | 1.05 | 0.96,1.14 | 1.06 | 0.97,1.16 |
| Smoking | Smoking | *Ref* | - | *Ref* | - | *Ref* | - |
|  | Quitting | 0.89** | 0.85,0.94 | 0.90** | 0.86,0.94 | 0.90** | 0.86,0.94 |
|  | Never smoking | 1.05** | 1.02,1.09 | 1.06** | 1.02,1.10 | 1.06** | 1.02,1.09 |
| Drinking | Drinking | *Ref* | - | *Ref* | - | *Ref* | - |
|  | Quitting | 1.06* | 1.00,1.13 | 1.06* | 1.00,1.13 | 1.06* | 1.00,1.13 |
|  | Never drinking | 1.08** | 1.04,1.12 | 1.10** | 1.06,1.14 | 1.08** | 1.05,1.12 |
| Having social activities or not | No | *Ref* | - | *Ref* | - | *Ref* | - |
|  | Yes | 0.95** | 0.92,0.98 | 0.94** | 0.91,0.97 | 0.94** | 0.92,0.97 |
|  | <6h | *Ref* | - | *Ref* | - | *Ref* | - |
| During of sleep | 6~9h | 0.74** | 0.72,0.76 | 0.73** | 0.71,0.75 | 0.73** | 0.71,0.75 |
|  | >9h | 0.72** | 0.67,0.77 | 0.72** | 0.67,0.77 | 0.71** | 0.66,0.76 |
| Chronic diseases | No chronic disease | *Ref* | - | *Ref* | - | *Ref* | - |
|  | Single chronic disease | 1.11** | 1.07,1.16 | 1.12** | 1.07,1.17 | 1.12** | 1.08,1.17 |
|  | Two or more chronic diseases | 1.27** | 1.23,1.32 | 1.31** | 1.26,1.36 | 1.31** | 1.26,1.36 |
| Self-reported health | Very good | *Ref* | - | *Ref* | - | *Ref* | - |
|  | Self-reported health | 1.00 | 0.98,1.02 | 1.00 | 0.99,1.02 | 1.00 | 0.98,1.02 |
| Health satisfaction | Dissatisfied | *Ref* | - | *Ref* | - | *Ref* | - |
|  | Satisfied | 1.00 | 0.97,1.04 | 1.00 | 0.97,1.04 | 1.00 | 0.97,1.04 |
| Life satisfaction | Dissatisfied | *Ref* | - | *Ref* | - | *Ref* | - |
|  | Satisfied | 0.99 | 0.95,1.03 | 0.99 | 0.95,1.03 | 0.98 | 0.94,1.03 |
|  | Dissatisfied | *Ref* | - | *Ref* | - | *Ref* | - |
| Marital satisfaction | Satisfied | 1.00 | 0.95,1.05 | 1.00 | 0.94,1.05 | 1.00 | 0.95,1.06 |
|  | No spouse | 1.01 | 0.94,1.09 | 1.01 | 0.94,1.09 | 1.02 | 0.95,1.11 |
| Child relationship satisfaction | Dissatisfied | *Ref* | - | *Ref* | - | *Ref* | - |
|  | Satisfied | 1.01 | 0.95,1.08 | 1.02 | 0.95,1.09 | 1.01 | 0.94,1.08 |
|  | No children | 1.03 | 0.85,1.26 | 1.04 | 0.85,1.27 | 1.02 | 0.84,1.24 |

Note: (1) All models included the individual, family, and community factors as random effects and adjusted for confounding factors of sex, age, household registration, marital status, education, ethnic groups, income, type of insurance, smoking, drinking, having social activities or not, during of sleep, chronic diseases, self-reported health, health satisfaction, life satisfaction, marital satisfaction, child relationship satisfaction. (2) *OR*: odds ratio; *CI*: confidence interval; *Ref*: reference. (3) **P*<0.05; ***P*<0.01.

**Table S3** Association of activities of daily living and depressive symptoms among the older adults (N=9641)

| Variables | Classification | Model 1 | | Model 2 | | Model 3 | |
| --- | --- | --- | --- | --- | --- | --- | --- |
|  |  | *OR_-unadjusted_* | 95% *CI* | *OR_-unadjusted_* | 95% *CI* | *OR_-unadjusted_* | 95% *CI* |
| - | (Intercept) | 0.51** | 0.48,0.54 | 0.59** | 0.55,0.63 | 0.55** | 0.51,0.58 |
| BADL | Unlimited | *Ref* | - | *Ref* | - | - | - |
|  | Limited | 2.31** | 2.04,2.62 | 3.58** | 3.19,4.03 | - | - |
| IADL | Unlimited | *Ref* | - | - | - | *Ref* | - |
|  | Limited | 2.42** | 2.17,2.71 | - | - | 3.39** | 3.06,3.74 |

Note: *OR*: odds ratio; *CI*: confidence interval; *Ref*: reference. (2) **P*<0.05; ***P*<0.01.

**Table S4** Association of daily living and depressive symptoms and its domains among older adults (N=9641)

| Variables | Classification | Model 4 | | Model 5 | | Model 6 | |
| --- | --- | --- | --- | --- | --- | --- | --- |
|  |  | *OR_-adjusted_* | 95% *CI* | *OR_-adjusted_* | 95% *CI* | *OR_-adjusted_* | 95% *CI* |
| - | (Intercept) | 0.42 | 0.28,0.65 | 0.45 | 0.30,0.69 | 0.44 | 0.29,0.67 |
| BADL | Unlimited | *Ref* | - | *Ref* | - | - | - |
|  | Limited | 1.92 | 1.69,2.19 | 2.70 | 2.39,3.05 | - | - |
| IADL | Unlimited | *Ref* | - | - | - | *Ref* | - |
|  | Limited | 2.10 | 1.87,2.36 | - | - | 2.68 | 2.41,2.98 |
| Sex | Male | *Ref* | - | *Ref* | *-* | *Ref* | - |
|  | Female | 1.06 | 0.96,1.17 | 1.05 | 0.95,1.16 | 1.07 | 0.96,1.18 |
| Age | 60-64 | *Ref* | - | *Ref* | - | *Ref* | - |
|  | 65-74 | 1.06 | 0.95,1.18 | 1.06 | 0.95,1.18 | 1.04 | 0.94,1.16 |
|  | ≥75 | 1.00 | 0.88,1.14 | 1.02 | 0.89,1.16 | 0.99 | 0.87,1.12 |
| Household registration | Urban | *Ref* | - | *Ref* | - | *Ref* | - |
|  | Rural | 1.02 | 0.90,1.15 | 1.00 | 0.89,1.14 | 1.01 | 0.89,1.14 |
| Marital status | Married and cohabiting | *Ref* | - | *Ref* | - | *Ref* | - |
|  | Separated | 1.04 | 0.81,1.32 | 1.02 | 0.80,1.30 | 1.02 | 0.80,1.30 |
|  | Divorced | 1.02 | 0.63,1.65 | 1.00 | 0.62,1.62 | 1.07 | 0.66,1.72 |
|  | Widowed | 1.00 | 0.85,1.19 | 1.01 | 0.85,1.20 | 1.00 | 0.84,1.18 |
|  | Never married | 1.04 | 0.52,2.10 | 0.98 | 0.49,1.98 | 1.00 | 0.50,2.00 |
| Education | Illiteracy | *Ref* | - | *Ref* | - | *Ref* | - |
|  | Primary school and below | 1.02 | 0.91,1.14 | 1.00 | 0.89,1.13 | 1.02 | 0.91,1.14 |
|  | Elementary school and above | 0.96 | 0.83,1.11 | 0.95 | 0.82,1.09 | 0.95 | 0.83,1.10 |
| Ethnic groups | Han | *Ref* | - | *Ref* | - | *Ref* | - |
|  | Non-Han | 0.99 | 0.80,1.21 | 0.99 | 0.81,1.21 | 1.00 | 0.81,1.22 |
| Income | Low | *Ref* | - | *Ref* | - | *Ref* | - |
|  | Middle | 0.93 | 0.84,1.02 | 0.92 | 0.83,1.02 | 0.93 | 0.85,1.03 |
|  | High | 0.98 | 0.83,1.16 | 0.97 | 0.82,1.14 | 0.99 | 0.84,1.17 |
| Type of insurance | Urban employees | *Ref* | - | *Ref* | - | *Ref* | -- |
|  | Urban and rural residents | 1.22 | 1.05,1.43 | 1.22 | 1.04,1.42 | 1.24 | 1.06,1.45 |
|  | Free medical care | 1.25 | 1.00,1.56 | 1.23 | 0.98,1.53 | 1.24 | 1.00,1.55 |
|  | Commercial insurance and others | 1.24 | 0.93,1.64 | 1.22 | 0.92,1.62 | 1.26 | 0.95,1.67 |
| Smoking | Smoking | *Ref* | - | *Ref* | - | *Ref* | - |
|  | Quitting | 0.82 | 0.70,0.95 | 0.82 | 0.71,0.95 | 0.83 | 0.72,0.97 |
|  | Never smoking | 1.17 | 1.05,1.31 | 1.19 | 1.06,1.32 | 1.18 | 1.06,1.32 |
| Drinking | Drinking | *Ref* | - | *Ref* | - | *Ref* | - |
|  | Quitting | 1.19 | 0.99,1.43 | 1.20 | 0.99,1.44 | 1.19 | 0.99,1.44 |
|  | Never drinking | 1.16 | 1.03,1.30 | 1.22 | 1.09,1.37 | 1.17 | 1.04,1.31 |
| Having social activities or not | No | *Ref* | - | *Ref* | - | *Ref* | - |
|  | Yes | 0.91 | 0.83,1.00 | 0.88 | 0.80,0.96 | 0.89 | 0.82,0.98 |
|  | <6h | *Ref* | - | *Ref* | - | *Ref* | - |
| During of sleep | 6~9h | 0.46 | 0.42,0.50 | 0.45 | 0.40,0.49 | 0.44 | 0.40,0.49 |
|  | >9h | 0.41 | 0.32,0.52 | 0.41 | 0.33,0.52 | 0.40 | 0.32,0.51 |
| Chronic diseases | No chronic disease | *Ref* | - | *Ref* | - | *Ref* | - |
|  | Single chronic disease | 1.31 | 1.14,1.51 | 1.36 | 1.18,1.56 | 1.34 | 1.17,1.54 |
|  | Two or more chronic diseases | 1.95 | 1.72,2.20 | 2.10 | 1.86,2.38 | 2.08 | 1.84,2.35 |
| Self-reported health | Very good | *Ref* | - | *Ref* | - | *Ref* | - |
|  | Self-reported health | 0.97 | 0.92,1.02 | 0.97 | 0.92,1.02 | 0.97 | 0.92,1.02 |
| Health satisfaction | Dissatisfied | *Ref* | - | *Ref* | - | *Ref* | - |
|  | Satisfied | 1.02 | 0.90,1.15 | 1.02 | 0.90,1.15 | 1.03 | 0.91,1.16 |
| Life satisfaction | Dissatisfied | *Ref* | - | *Ref* | - | *Ref* | - |
|  | Satisfied | 0.97 | 0.85,1.12 | 0.98 | 0.85,1.12 | 0.96 | 0.83,1.10 |
|  | Dissatisfied | *Ref* | - | *Ref* | - | *Ref* | - |
| Marital satisfaction | Satisfied | 1.02 | 0.86,1.21 | 1.00 | 0.84,1.19 | 1.03 | 0.87,1.23 |
|  | No spouse | 1.09 | 0.84,1.41 | 1.06 | 0.82,1.37 | 1.11 | 0.86,1.44 |
| Child relationship satisfaction | Dissatisfied | *Ref* | - | *Ref* | - | *Ref* | - |
|  | Satisfied | 1.10 | 0.88,1.38 | 1.13 | 0.90,1.41 | 1.08 | 0.87,1.35 |
|  | No children | 1.05 | 0.55,2.03 | 1.10 | 0.57,2.11 | 1.03 | 0.54,1.97 |

Note: (1) All models included the individual, family, and community factors as random effects and adjusted for confounding factors of sex, age, household registration, marital status, education, ethnic groups, income, type of insurance, smoking, drinking, having social activities or not, during of sleep, chronic diseases, self-reported health, health satisfaction, life satisfaction, marital satisfaction, child relationship satisfaction. (2) *OR*: odds ratio; *CI*: confidence interval; *Ref*: reference. (3) **P*<0.05; ***P*<0.01.
